# SRW-YOLOv8n: a high-precision method for main-stem detection and clamping-point positioning of plug pepper seedlings

**DOI:** 10.3389/fpls.2026.1789467

**Published:** 2026-06-11

**Authors:** Jiang Li, Mingshuo Ding, HuiMin Liu, Jingye Xu, Laixiang Liu, Jiasheng Wang

**Affiliations:** 1 College of Mechanical and Electrical Engineering, Qingdao Agriculture University, Qingdao, Shandong, China; 2 Shandong Provincial Key Laboratory of Smart Agricultural Equipment for Protected Horticulture, Qingdao, Shandong, China

**Keywords:** clamping-point, deep learning, pepper grafting machine, plug pepper seedlings, shielding-supporting mechanism, SRW-YOLOv8n

## Abstract

Precise positioning of clamping-points is the core and difficulty of realizing fully automated grafting of plug pepper seedlings. Traditional mechanical positioning methods often struggle to accommodate the morphological variations of pepper seedlings across an entire plug tray, resulting in large positioning errors and high clamping failure rates. To address this problem, this study develops an improved YOLOv8n-based framework for accurate detection and spatial positioning of seedling clamping points. The baseline YOLOv8n is optimized by integrating the SimAM, RFAConv and WIoU loss function to establish an enhanced SRW-YOLOv8n model. Moreover, a shielding-supporting mechanism and structured image processing strategy are adopted to suppress dense seedling interference, and depth camera calibration is applied to convert pixel coordinates into 3D spatial coordinates. Experimental results show that the SRW-YOLOv8n achieves 96.6% precision, 98.4% recall, 97.5% F1 and 97.4% mAP@0.5, outperforming the original YOLOv8n. The proposed system delivers average absolute positioning errors of 2.49 mm, 2.39 mm and 1.83 mm in the x, y and z axes, fully satisfying high-precision grafting requirements. This method provides robust spatial positioning guidance for automated pepper seedling grafting operations.

## 1 Introduction

As an important condiment crop widely cultivated worldwide, pepper has increasingly been produced using intensive and facility-based cultivation systems in recent years (Zhang et al., 2024; Liang et al., 2024; Alonso-Villegas et al., 2023). Among these, greenhouse cultivation has been widely adopted, as it allows for effective regulation of the growing environment, thus shortens the growth cycle, and enables off-season production (Lei et al., 2023). This cultivation mode improves production efficiency and enhances economic returns (Cui et al., 2022; Li et al., 2023; Zeeshan Ul Haq et al., 2023). However, this cultivation model inevitably leads to continuous cropping problems. Grafting can improve plant resistance, reduce pests and diseases incidence, and increase crop yield by complementing the strengths of two plants. It thus overcomes the harmful effects of continuous cropping (Nie and Wen, 2023; Yan et al., 2022; Chen et al., 2024). However, the plug pepper seedlings used in mechanical grafting exhibit dense spatial distribution, mutual occlusion, and significant morphological variability. These characteristics limit the effectiveness of traditional fixed-trajectory clamping mechanisms. As a result, traditional mechanical positioning grafting machines struggle to achieve adaptive clamping, leading to a high clamping failure rate (Li et al., 2024). Therefore, the precise positioning of seedling clamping points and the adaptive clamping are crucial for realizing fully automated grafting of plug pepper seedlings (Wang et al., 2022).

In recent years, with the development of agricultural production toward higher efficiency, standardization, and automation, machine vision technology has been widely applied in various stages of agricultural engineering. By employing algorithms such as object detection, image processing, and image localization, this technology enables accurate recognition, information acquisition, and spatial positioning of agricultural targets (Wang and Liu, 2024; Ruchay et al., 2024; Deng et al., 2025).

In terms of object detection, convolutional neural network (CNN)-based algorithms are primarily used. These algorithms are divided into single-stage algorithms, such as YOLO and SSD, and two-stage algorithms, such as R-CNN series, based on whether candidate regions are generated during the detection process. Both types have their own advantages and disadvantages in terms of speed and accuracy. They are widely applied in object detection. For example, Li proposed an automatic hydroponic lettuce seedling detection algorithm based on an improved Faster R-CNN (Li et al., 2021). By adopting HRNet as the backbone network and replacing the pooling layer with RoI Align, the algorithm optimizes feature extraction. The improved network achieved an mAP of 86.2% for hydroponic lettuce seedlings. Schneider conducted a study on full life-cycle detection of pepper plants based on YOLOv8 (Schneider et al., 2024). By training and comparing models using datasets composed of different combinations of viewing angles, they found that the combination of top-down and side views achieved the best detection performance, with a detection accuracy of up to 96.3%.

In terms of image processing, the focus is on analyzing and processing images. The goal is to improve the visual effects, enhance the image quality, or extract useful information. For example, Suarez developed a Python−based system using OpenCV to measure citrus leaf area (Suarez et al., 2025). The system acquires and preprocesses images. It converts them to HSV space and segments leaves from the background using adaptive thresholds. Contour extraction and morphological filtering are applied to detect leaf boundaries and automatically calculate leaf area. Jin developed a seedling picking-point estimation algorithm (Jin et al., 2024). The algorithm applies a series of image processing operations to locate seedling leaves and calculate the optimal picking angle for a mechanical gripper.

In terms of image matching and positioning, the main principle is to simulate binocular disparity to perceive depth. By combining triangulation with multiple lenses capturing images of the same object from different angles, image matching can be used for precise distance measurement. For example, Hu developed a strawberry picking robot system using YOLO and Mask R−CNN for 2D detection and segmentation (Hu et al., 2022). A 3D binocular camera obtains depth data. The system integrates depth with 2D coordinates to calculate each strawberry’s 3D position. This enables precise grasping and harvesting. Yang applied an improved YOLOv8 algorithm to detect grape picking points based on keypoint detection (Yang et al., 2024). A depth camera was then used to acquire the three-dimensional real-world coordinates of the picking points in the camera coordinate system.

However, existing studies mainly focus on general object detection or harvesting applications, and relatively few studies have addressed the precise localization of clamping points for plug pepper seedlings under the dense growth conditions. In particular, challenges such as occlusion among seedlings, morphological variability across plug trays, and the integration of detection results with high-precision spatial positioning remain insufficiently explored.

Building on these methods, this study presents a spatial positioning method for clamping-points of plug pepper seedlings using the SRW-YOLOv8n object detection model. The method improves the YOLOv8 model to construct a new detection model, SRW-YOLOv8n, which enables high-precision detection of the main stems of plug pepper seedlings. In addition, an occlusion-support device is designed to enhance detection accuracy. Pixel coordinates of the clamping-point are then extracted through structured image processing. Finally, using a depth camera and coordinate transformation, these coordinates are mapped to the camera coordinate system, yielding the 3D spatial coordinates of the clamping-point. Through this approach, precise spatial localization of clamping-points is achieved, providing accurate 3D spatial coordinate guidance for subsequent automated clamping operations.

## 2 Materials

### 2.1 Dataset acquisition

**Figure 1:**
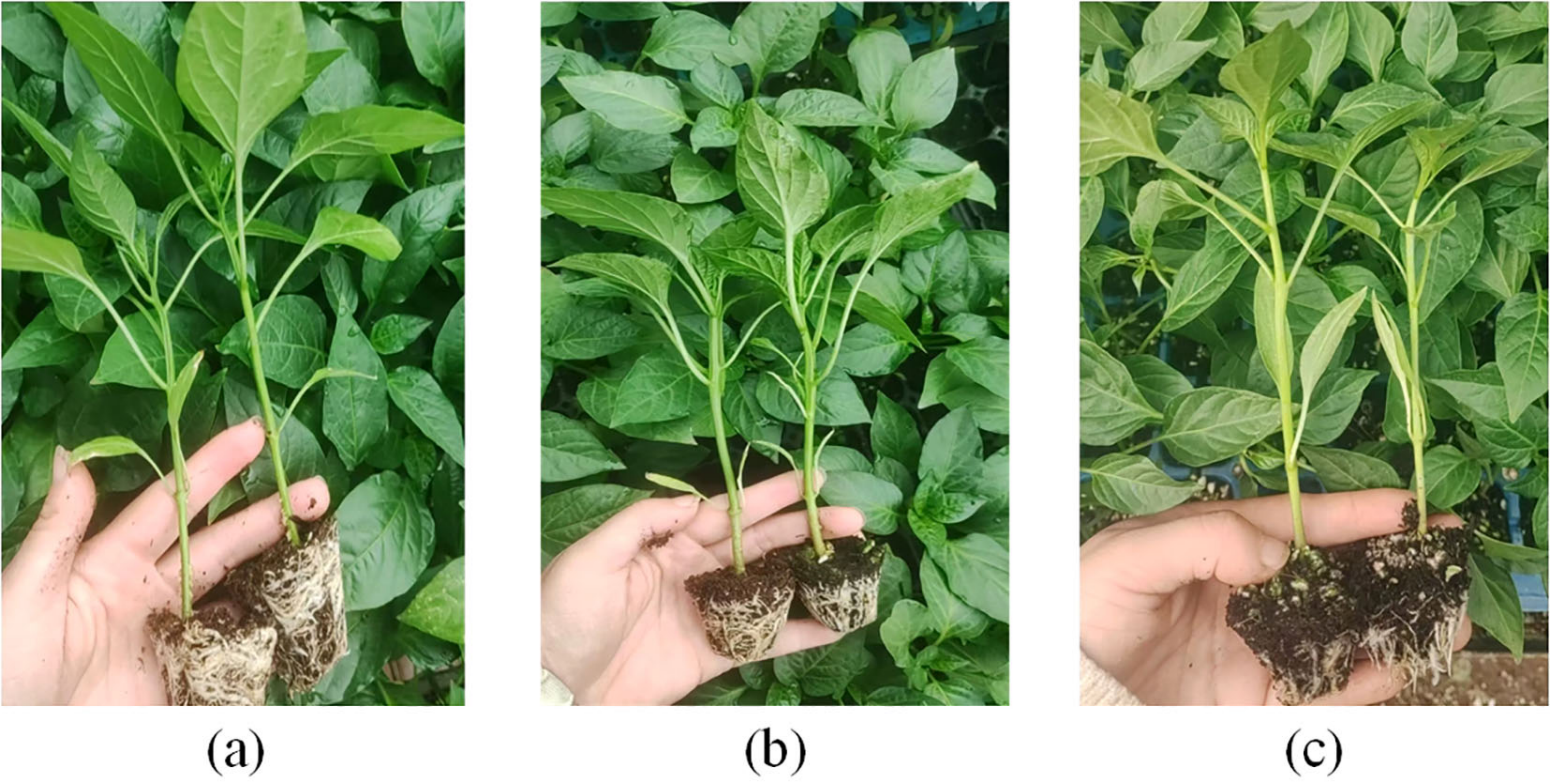
Three commonly cultivated pepper varieties. **(a)** Thin-skinned Green Pepper No.1; **(b)** Hangjiao Pepper No.1; **(c)** Line Pepper No.336.

To construct a high-quality dataset suitable for main-stem detection of plug pepper seedlings and to ensure the model’s generalization capability in complex environments, this study systematically collected images of seedlings in the laboratory of Qingdao Agricultural University. The dataset included three commonly cultivated pepper varieties—Thin-skinned Green Pepper No.1, Hangjiao Pepper No.1, and Line Pepper No.336. The three varieties were sampled in approximately balanced proportions, as shown in **Figure 1**. The three varieties are morphologically similar in the early seedling stage, with the main differences being the height of the base of the cotyledons from the substrate and the thickness of the main stem. Images were captured over a period of 20 to 30 days after sowing, during which the stem diameter of different seedlings increased by approximately 30–50%, thereby increasing the richness of the dataset for main-stem detection within the grafting period. During acquisition, the seedlings were arranged naturally, reflecting normal growth conditions with typical occlusion and overlap among plants, to represent realistic growing scenarios. In total, 500 valid images were obtained.

### 2.2 Dataset preprocessing

**Figure 2:**
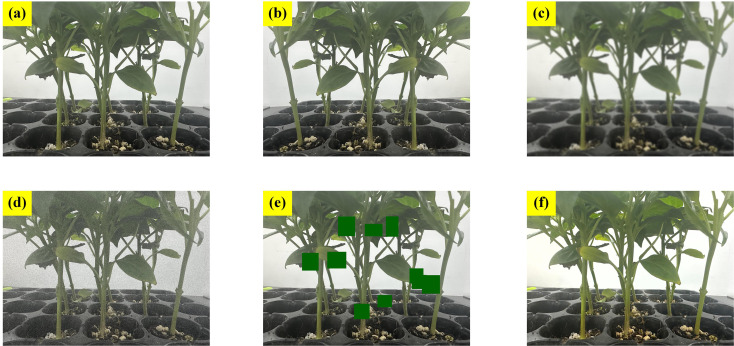
Data augmentation. **(a)** Original image; **(b)** Turn left and right; **(c)** Gaussian blur; **(d)** Salt and pepper noise; **(e)** Random occlusion; **(f)** Brightness change.

In deep learning training, data scarcity is one of the most common challenges in model development. A dataset that is too few may lead to overfitting in training, while constructing a sufficiently large and high-quality dataset is often time-consuming and labor-intensive. Data augmentation provides a cost-effective and efficient solution for dataset expansion. Typical augmentation techniques include noise addition, brightness adjustment, flipping, and rotation (Suto, 2024). In this study, strictly adhering to the fundamental principles of no missed labels and no duplicate labels, each image was manually annotated using the Labelme tool. This process generated the corresponding label files. Subsequently, five augmentation methods, as shown in **Figure 2**, were applied to each image using the PIL and OpenCV libraries. This generated new images along with their corresponding labels. This process expanded the dataset to 3,000 images with labels, thus completing the dataset construction.

### 2.3 Dataset partitioning

According to prior research conducted both previous studies, the partition ratio of a dataset can significantly affect the training performance of deep learning models, and the optimal partitioning scheme varies across different datasets (Sunoj et al., 2018). Based on commonly used partition ratios reported in previous studies on deep learning for object detection, four dataset partitioning schemes were designed for this study, as shown in **Table 1**.

**Table 1:** Distribution of data sets with four division ratios.

Group	Partition ratio	Training set	Validation set	Test set
a	8:1:1	2400	300	300
b	7:2:1	2100	600	300
c	6:3:1	1800	900	300
d	6:2:2	1800	600	600

During dataset partitioning, all augmented images derived from the same original image were treated as a single group and assigned exclusively to the same subset (training, validation, or test set). This group-wise splitting strategy ensures that no near-duplicate or augmented “sibling” samples appear across different subsets, thereby effectively preventing potential data leakage.

## 3 Methods

### 3.1 YOLOv8 model

YOLOv8 is one of the more mature versions of the YOLO series. It has high detection efficiency, a simple structure, and strong deployment performance. It has been widely used in agricultural object detection tasks. Compared with earlier models, YOLOv8 retains the advantages of the YOLO series while introducing several improvements. These include an enhanced backbone network and dynamic label assignment strategies, which further improve detection accuracy and efficiency. Compared with later versions, YOLOv8 has a more stable and mature network architecture. It also offers excellent adaptability for deployment. The model supports seamless export to mainstream inference frameworks such as ONNX and TensorRT. In addition, it provides lightweight deployment solutions for mobile devices, significantly enhancing convenience and compatibility in practical agricultural applications.

YOLOv8 comprises five model variants: YOLOv8n, YOLOv8s, YOLOv8m, YOLOv8l, and YOLOv8x. To ensure a lightweight design and facilitate subsequent deployment, this study employs the YOLOv8n version, which has the smallest number of parameters. In the YOLOv8 model structure, the backbone is responsible for feature extraction, which the C2f module used as the basic unit to replace the C3 module in YOLOv5. This replacement reduces redundant parameters and improves computational efficiency through structural optimization. The neck is responsible for multi-scale feature fusion. It adopts a path aggregation network structure to enhance the model’s ability to integrate features across different scales. The head employs a decoupled head structure and an anchor-free strategy. Three detection heads operate at different scales, ultimately outputting target categories and bounding box information.

### 3.2 SRW-YOLOv8n model

**Figure 3:**
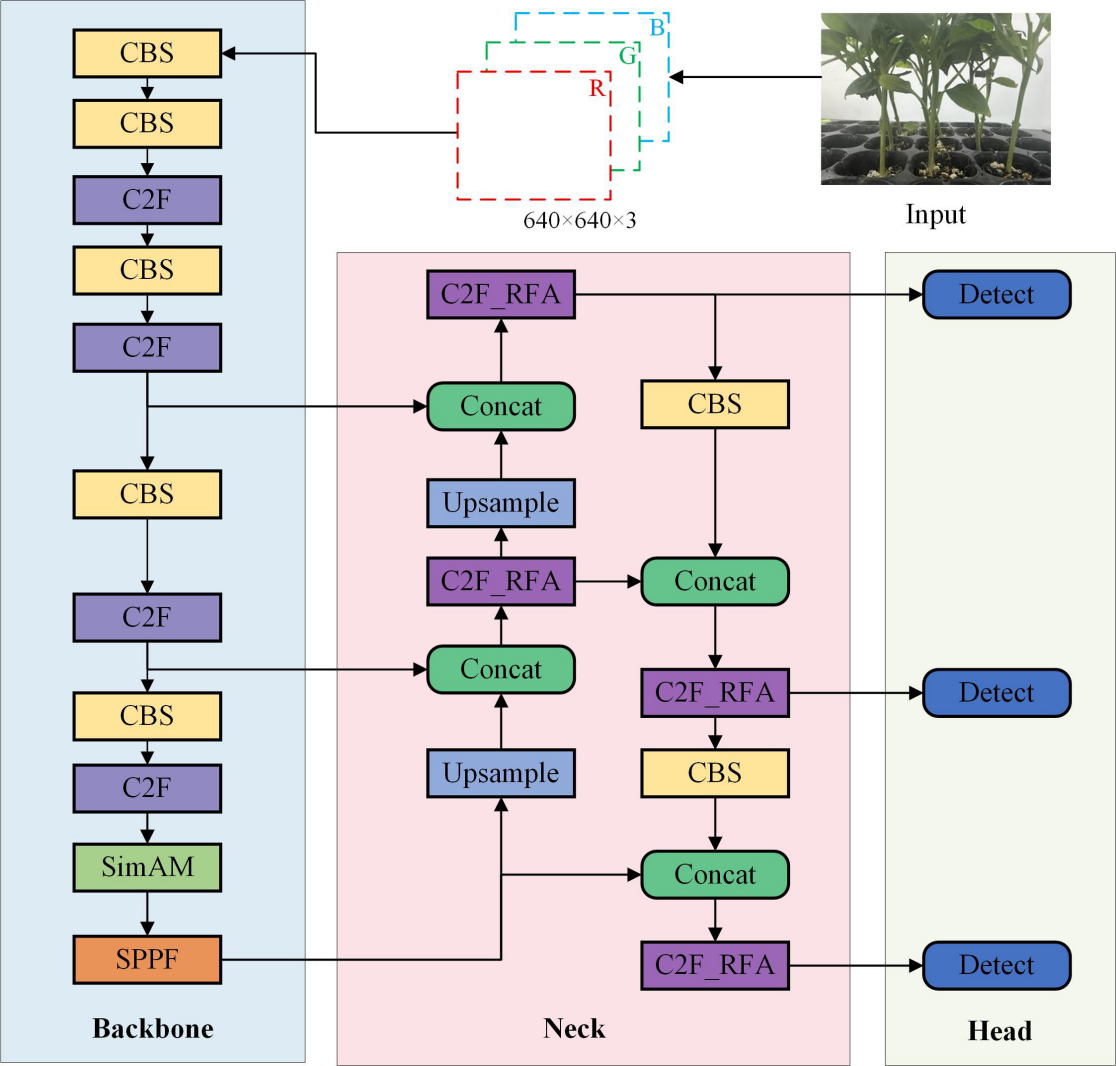
SRW-YOLOv8n network structure.

Plug pepper seedlings are densely arranged and often occlude each other, which makes main-stem recognition challenging. Therefore, this study constructed an improved model, SRW-YOLOv8n, based on YOLOv8n. The overall structure of the improved model is shown in **Figure 3**. First, the Simple Attention Module (SimAM) was introduced into the neck network (Yang et al., 2021). It adaptively enhances key features using a neuron energy function while effectively suppressing noise. Second, the second standard convolution in the C2f module of the neck was replaced with Receptive Field Attention Convolution (RFAConv) (Zhang et al., 2023). This replacement reduces model complexity and computational cost while improving spatial perception. Finally, the Wise-IoU (WIoU) loss function (Tong et al., 2023) was adopted to replace the original Complete-IoU (CIoU) loss. This change improves the matching accuracy between predicted and ground-truth bounding boxes.

#### 3.2.1 SimAM: a simple attention module

**Figure 4:**
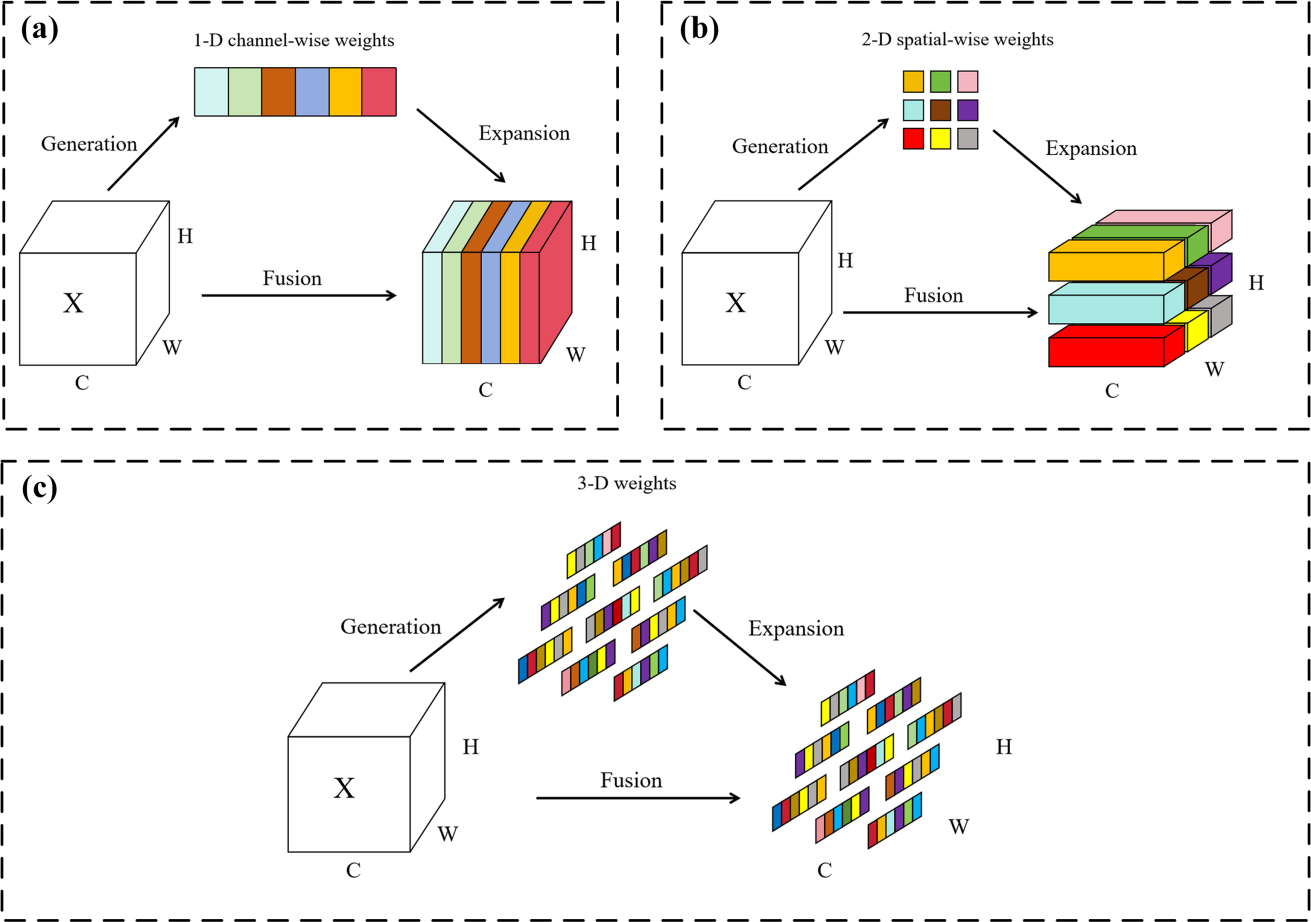
Structure of attention channel. **(a)** Channel attention mechanisms; **(b)** Spatial attention mechanisms; **(c)** SimAM attention mechanism.

Traditional attention mechanisms enhance important features while suppressing irrelevant ones by assigning weights to different dimensions of feature maps. As shown in **Figures 4a, b**, they can be classified into two categories based on the dimensionality and the target of the weights: channel attention mechanisms, which use one-dimensional weights, and spatial attention mechanisms, which use two-dimensional weights. However, in the main-stem detection task for dense plug pepper seedlings, conventional attention mechanisms have limitations. Traditional attention mechanisms compute channel and spatial weights separately, which limits their ability to simultaneously capture the spatial–channel correlations critical for highlighting key stem features.

To address the challenges of densely arranged plug pepper seedlings, the Simple Attention Module (SimAM) was introduced before the SPPF module. As shown in **Figure 4c**, SimAM is a parameter-free three-dimensional attention mechanism inspired by the human brain’s attention mechanism. It integrates both channel and spatial information to evaluate the importance of each neuron in the feature map using an energy function, enhancing neuron-level feature representation without increasing computational parameters. The core of SimAM lies in defining an energy function to evaluate the importance of each position (neuron) in the feature map. The formula for calculating the minimum energy *e_t_* of a neuron relative to other neurons in the same channel is given in Equation 1:

(1) $$e_{t}=\frac{4(\sigma^{2}+\lambda )}{{\left(t-\mu \right)}^{2}+2\sigma^{2}+2\lambda }$$

Where, *t* represents the target neuron, *λ* represents the regularization coefficient, *σ*^2^ represents the variance of the feature values of all neurons in the neighborhood of the target neuron, and *μ* represents the mean value of the feature values of all neurons in the neighborhood of the target neuron.

The lower the energy *e_t_*, the more distinct the neuron is from others, reflecting its greater importance. By computing and assigning weights to each neuron, the network’s feature discrimination ability and overall recognition performance can be effectively enhanced. The importance-weighting formula of neurons in the SimAM module is shown in Equation 2:

(2) $$X_{1}=sigmoid(\frac{1}{E})⊙X_{2}$$

Where, *E* represents the energy across all channel and spatial dimensions, *X*_1_ represents the enhanced feature map, *X*_2_ represents the input feature map, and ⊙ represents the matrix multiplication operator. The *sigmoid* activation function constrains excessively large values in *E*, thereby enhancing the relative importance among neurons.

#### 3.2.2 RFAConv: a receptive field attention convolution

The RFAConv is a novel convolution module. Unlike traditional convolutions with fixed kernel sizes, RFAConv can dynamically adjust the kernel size according to feature-specific requirements. This enhances spatial information perception. Compared with conventional attention mechanisms, RFAConv not only maintains high performance but also significantly improves learning efficiency.

**Figure 5:**
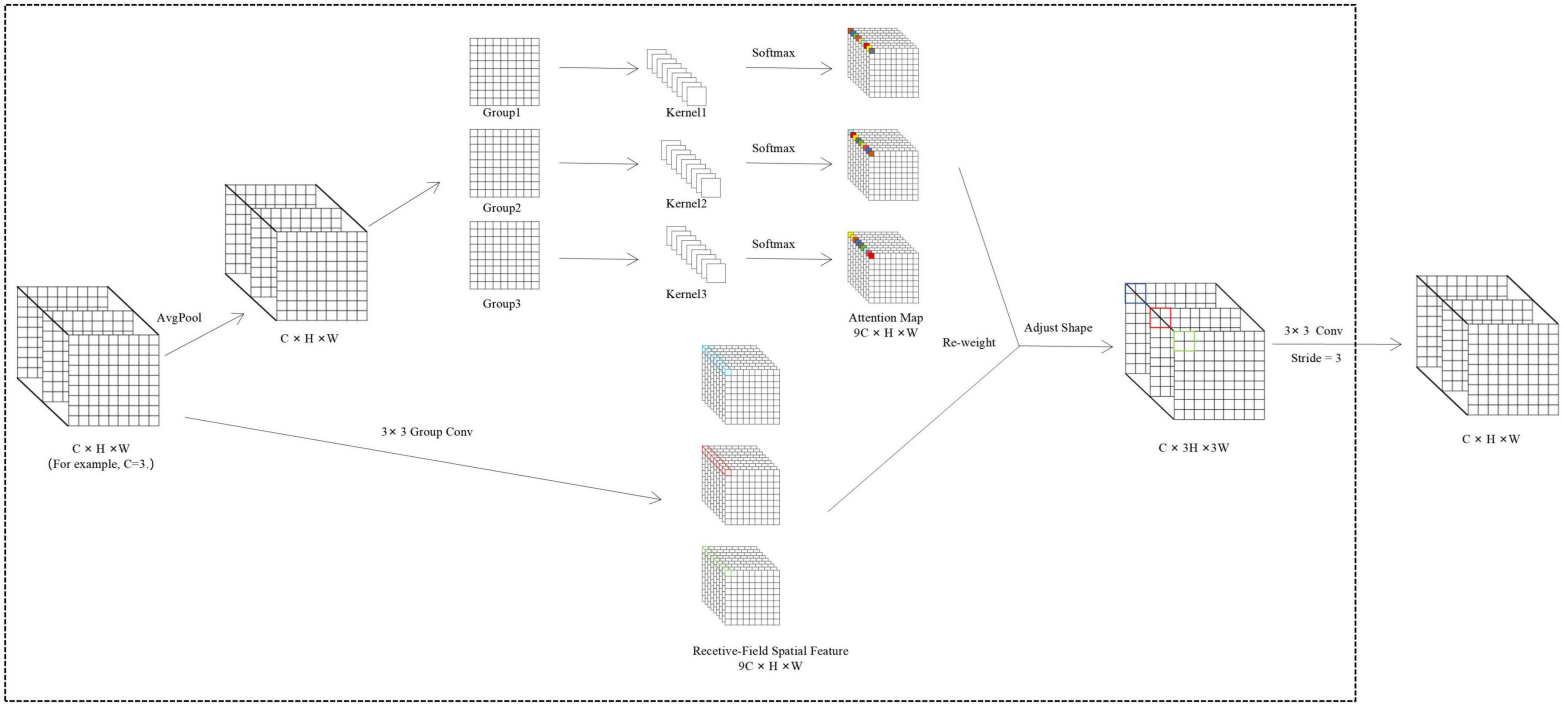
RFAConv operation process.

As shown in **Figure 5** (taking the case of input channels *C* = 3 as an example), the RFAConv module contains two branches: a weight generation branch and a feature branch. In the weight generation branch, the global information of each receptive field feature is first aggregated through average pooling without changing the number of channels *C*. A 1×1 convolution is then applied to divide the features into *C* independent groups, with each group containing only one channel to enable independent channel processing. Next, grouped convolution is used to expand the output channels of each group to *k*². This not only reduces computational cost but also enables effective information interaction among features of different groups. Subsequently, the *Softmax* function is applied to assign weights to the feature information within each receptive field, resulting in the attention map *A_rf_*. In the feature branch, the original input feature map undergoes a standard *k*×*k* convolution operation. It is then normalized and activated to obtain the transformed receptive field feature *F_rf_*. Finally, the receptive field features are weighted according to the attention map and adjusted to the appropriate size, yielding the output of the Receptive-Field Attention Convolution. The computational process of RFAConv is shown in Equation 3:

(3) $$F=Softmax(g^{1\times 1}(\text{AvgPool}(X)))\times ReLU(\text{Norm}(g^{k\times k}(X)))=A_{rf}\times F_{rf}$$

Where, *g*^1×1^ represents a grouped convolution with a kernel size of 1×1, *X* represents the input feature map, *g^k^*^×^*^k^* represents a convolution with a kernel size of *k*×*k*, and *F* represents the output feature map.

**Figure 6:**
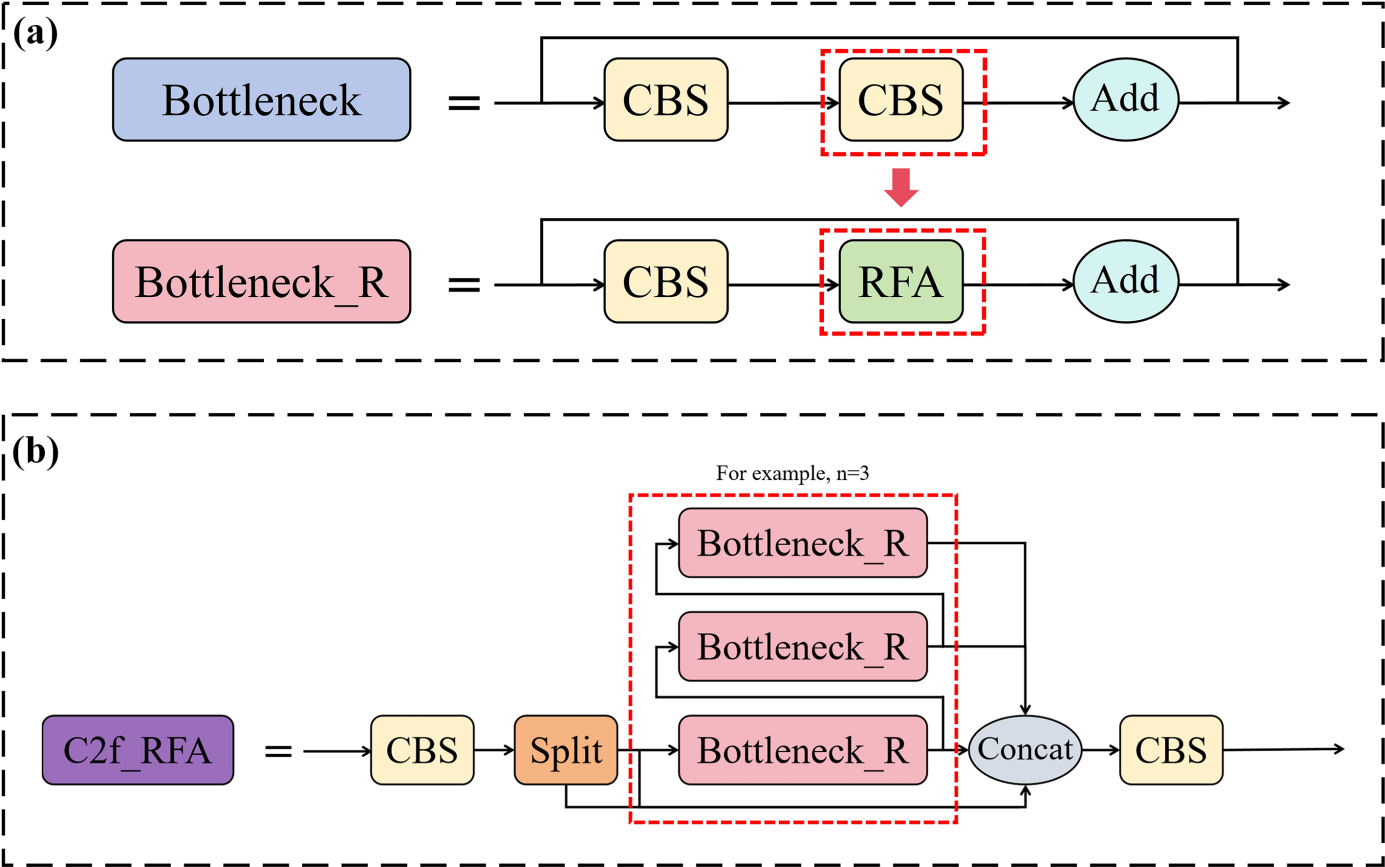
Module improvements. **(a)** Bottleneck_R structure; **(b)** C2f_RFA module structure.

In the neck network structure of YOLOv8n, a large number of C2f modules are employed. These modules enrich feature representations through multi-branch structures and feature concatenation, thereby improving the overall network performance. Therefore, the performance of the entire network is closely related to the feature learning capability of the C2f modules. However, in the main-stem detection task for plug pepper seedlings, the stems are slender and frequently occluded by leaves. Under such conditions, fixed convolution kernels may respond too strongly to surrounding leaf textures or background noise, thereby affecting the discriminative representation of stem regions. To address this issue, the C2f modules in the neck network of YOLOv8n were improved in this study. As shown in **Figure 6a**, the second standard convolution in the Bottleneck module was replaced with an RFAConv module, resulting in the C2f_RFA module shown in **Figure 6b**. By introducing receptive-field-level adaptive weighting, the improved module strengthens the representation of slender stem features while maintaining a lightweight structure. This modification effectively enhances the network’s feature modeling of key regions without significantly increasing the number of model parameters, thereby improving main-stem detection accuracy.

#### 3.2.3 WIoU loss function

**Figure 7:**
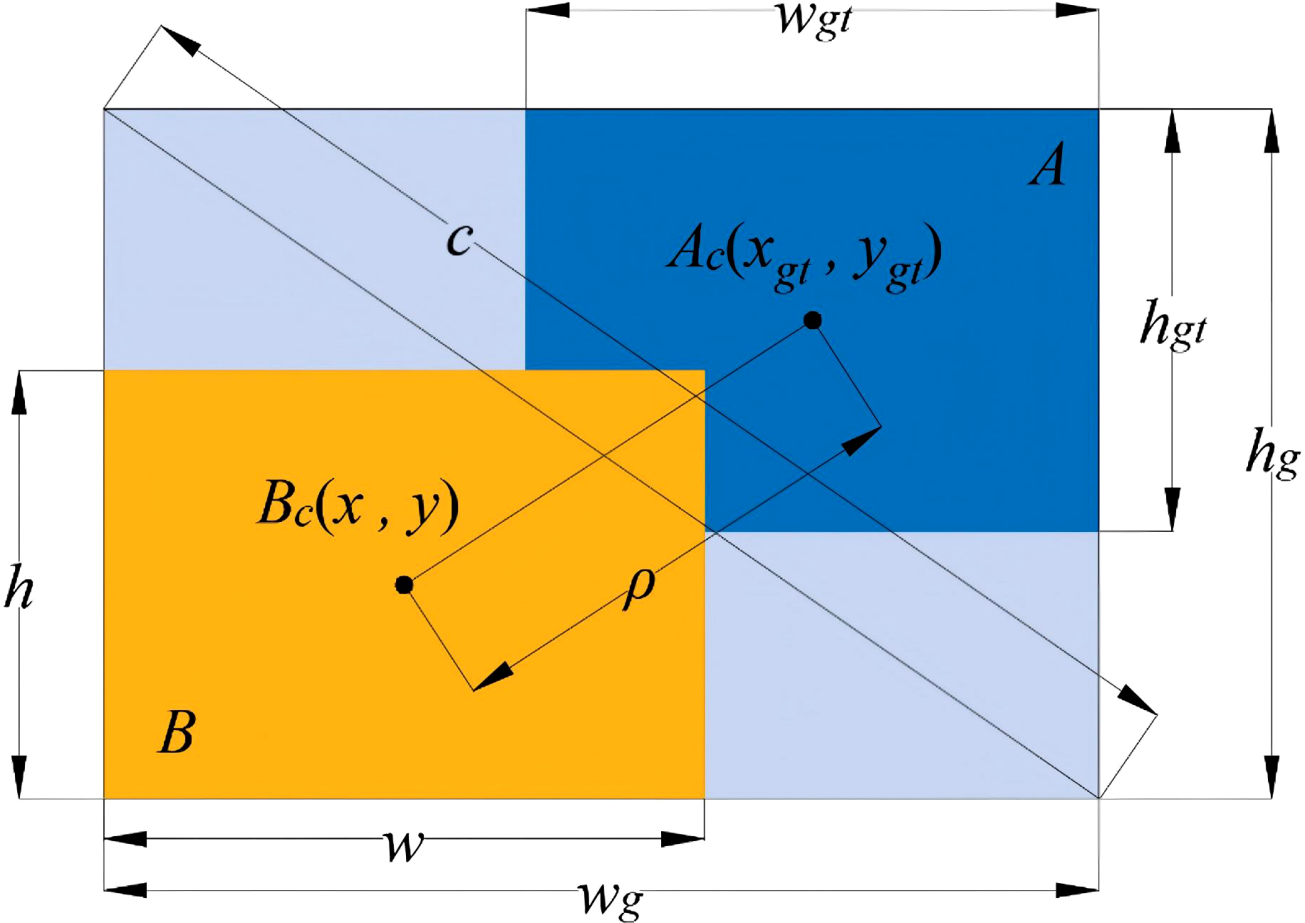
Schematic diagram of geometric relationship between real box and prediction box. *A_c_*(*x*,*y*) represents the center position of the predicted bounding box, *B_c_*(*x_gt_*,*y_gt_*) represents the center position of the ground-truth bounding box, *w_g_* and *h_g_* represent the width and height of the minimum enclosing rectangle covering both the ground-truth and predicted bounding boxes, *w* and *h* represent the width and height of the predicted bounding box, *w_gt_* and *h_gt_* represent the width and height of the ground-truth bounding box.

Loss functions are a core component of deep learning model training. Their primary role is to quantify the difference between model predictions and ground truth values. By computing gradients, the loss function provides parameter update directions for optimization algorithms such as stochastic gradient descent and Adam. This process guides iterative optimization of model parameters and drives the model toward an optimal solution. A well-designed loss function can balance model convergence speed and generalization ability, enabling the model to maintain stable prediction performance even in complex scenarios. As shown in Equation 4, the CIoU loss function used in the traditional YOLOv8 model introduces center-point distance constraints and aspect-ratio consistency constraints based on IoU. Compared with earlier loss functions that rely only on IoU, CIoU further improves bounding box localization accuracy. It performs particularly well in scenarios with blurred boundaries or overlapping targets. However, as shown in Equations 5–8, when the predicted box and the ground-truth box have the same aspect ratio, the aspect-ratio consistency penalty term *v* is always zero (the geometric relationship is shown in **Figure 7**). This partially masks size discrepancies, significantly reducing the sensitivity of IoU-based loss functions to scale errors, and thereby weakening the model’s ability to learn the absolute size of the target.

(4) $$L_{CIoU}=L_{IoU}+\frac{\rho^{2}(A_{c},B_{c})}{c^{2}}+\varphi v$$

Where:

(5) $$L_{IoU}=1-IoU(A,B)$$

(6) $$IoU=\frac{A\cap B}{A\cup B}$$

(7) $$\varphi =\frac{v}{L_{IoU}+v}$$

(8) $$v=\frac{4}{\pi^{2}}{\left(arctan\frac{w^{gt}}{h^{gt}}-arctan\frac{w}{h}\right)}^{2}$$

Where, *L_CIoU_* represents the CIoU loss function, *L_IoU_* represents the IoU loss function, *A* represents the ground-truth bounding box, *B* represents the predicted bounding box, *IoU*(*A,B*) represents the intersection-over-union between *A* and *B*, *ρ*(*A_c_*,*B_c_*) represents the Euclidean distance between the centers of the ground-truth and predicted bounding boxes, *c* represents the diagonal length of the smallest enclosing rectangle covering both the ground-truth and predicted boxes, *φ* represents the weighting factor, and *v* represents the aspect ratio penalty term between the ground-truth and predicted bounding boxes.

In the main-stem detection task for plug pepper seedlings, the annotated bounding boxes of seedlings within the same batch usually have similar aspect ratios due to the similar morphology of the seedlings’ main stems. During training, the predicted bounding boxes therefore tend to have the same or very similar aspect ratios as the ground-truth boxes. In the CIoU loss function, this situation causes the penalty term *v* to degenerate, which weakens the model’s ability to constrain target scale deviations and, consequently, degrades the overall training performance. This study introduced an optimized loss function WIoU to address this issue. It removed the penalty term *v* in CIoU and incorporated an explicit size penalty term, R_WIoU_. This fully overcomes the problem in where the loss function fails to optimize when the predicted and ground-truth boxes have identical aspect ratios. WIoU is a bounding box regression loss function with a dynamic focusing mechanism, which optimizes the training process through a gradient gain modulation strategy. The WIoU series includes three progressive versions, among which version v3 achieves the best performance. Its calculation formula is given in Equations 9–12.

(9) $$L_{WIoU\_\text{v}3}=r\cdot R_{WIoU}\cdot L_{IoU}$$

Where:

(10) $$r=\frac{\beta }{\delta \alpha^{\beta -\delta }}$$

(11) $$\beta =\frac{L_{Io}^{U^{*}}}{\bar{L_{IoU}}}\in [0,+\infty )$$

(12) $$R_{WIoU}=\exp(\frac{{\left(x-x_{gt}\right)}^{2}+{\left(y-y_{gt}\right)}^{2}}{{\left(w_{g}^{2}+h_{g}^{2}\right)}^{*}})$$

Where, *L_WIoU__*_v3_ represents the loss function of WIoU_v3, *r* represents the non-monotonic focusing factor, *β* represents the outlier degree, *δ* represents the gain coefficient, *α* represents the learning factor, and _*_ represents the decoupling of *w_g_* and *h_g_* from the computation graph.

### 3.3 Clamping-point pixel localization based on structured image processing

**Figure 8:**
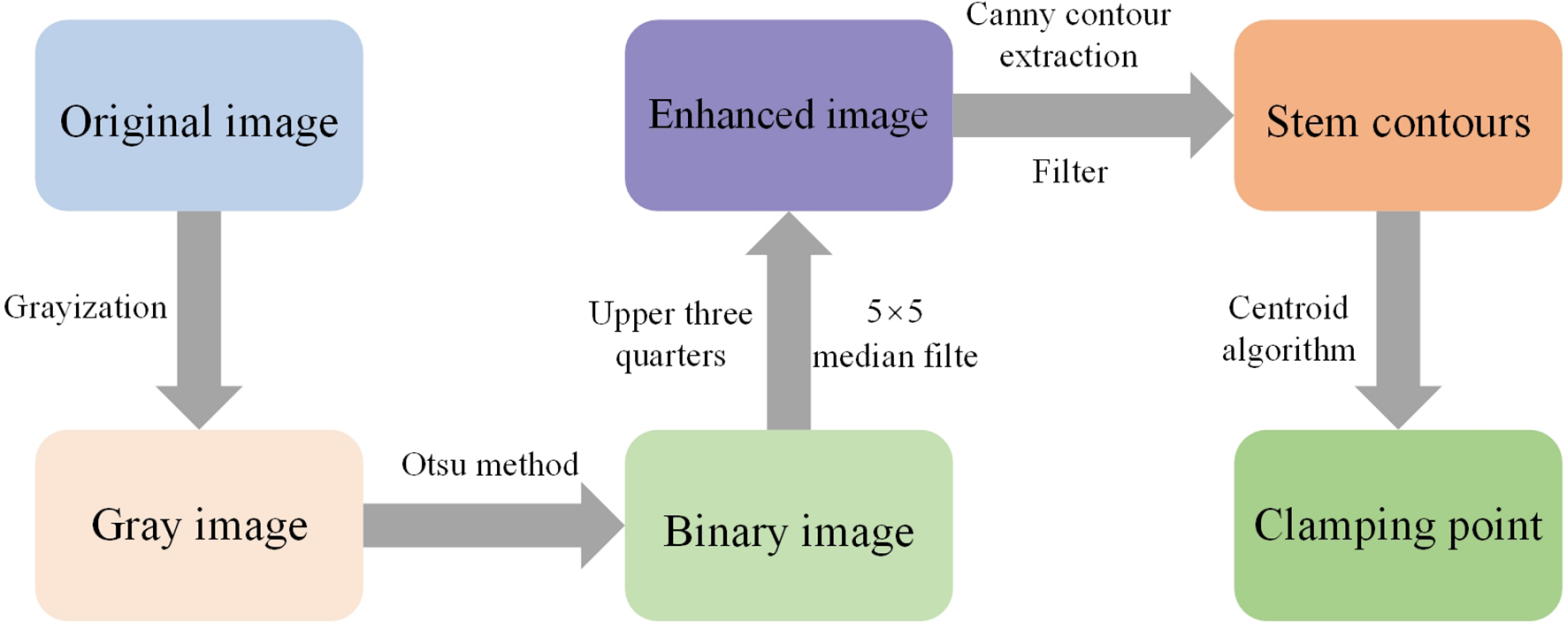
Structured image processing workflow.

To precisely localize the pixel coordinates of clamping-points in plug pepper seedlings, this study utilized the optimal weights obtained from training the SRW-YOLOv8n model to detect the main stems. The bounding boxes of the detected stem regions are then extracted and processed through a structured image processing workflow, as shown in **Figure 8**. Specifically, the RGB image was first converted to grayscale image to reduce computational complexity. Then, Otsu’s method was applied to determine the global optimal threshold, followed by image binarization to enhance contrast between the stem region and the background. A 5×5 median filter was subsequently applied to the binary image to suppress isolated noise points and remove lateral branches of the stem. During edge detection, to avoid interference from the gap between the baffle and the plug tray on contour extraction, only the upper three-quarters of the image are retained. The Canny operator is then applied to extract the stem contour, and the contour region is filtered to retain only the outermost stem contour. Finally, the centroid algorithm was used to calculate the geometric center of the main contour as the clamping-point for seedling picking. This point is then transformed from the local bounding box coordinate system to the original image coordinate system, yielding the pixel coordinates of the clamping-point in the original image.

### 3.4 3D coordinate mapping and distortion correction of clamping-points

In real-world scenarios, camera lenses cannot fully conform to the optical assumptions of the ideal pinhole camera model. To increase light intake and ensure imaging quality, practical cameras typically use complex multi-element lens systems. However, the inherent physical properties of these lenses, combined with assembly deviations, cause non-ideal refraction of incident light rays, ultimately leading to image distortion. Therefore, before performing coordinate transformation, distortion correction must be applied to both the color image and the depth map using the distortion coefficients. After the correction is completed, the pixel coordinates of the target point are mapped to the three-dimensional spatial coordinates in the camera coordinate system by combining the pixel coordinates with depth information and using a coordinate transformation method. The specific transformation formula is shown in Equations 13–15:

(13) $$\text{x}=\frac{(u-c_{x})\cdot d}{f_{x}}$$

(14) $$y=\frac{(v-c_{y})\cdot d}{f_{y}}$$

(15) z=d

Where, *f_x_* and *f_y_* represent the focal lengths of the camera along the x-axis and y-axis; *c_x_* and *c_y_* represent the coordinates of the camera’s principal point; *d* represents the depth value corresponding to the pixel; (*u*, *v*) represents the pixel coordinates of the target point; and (*x*, *y*, *z*) represents the three-dimensional spatial coordinates of the target point.

### 3.5 Test bench for precise positioning of pepper seedling clamping-points

**Figure 9:**
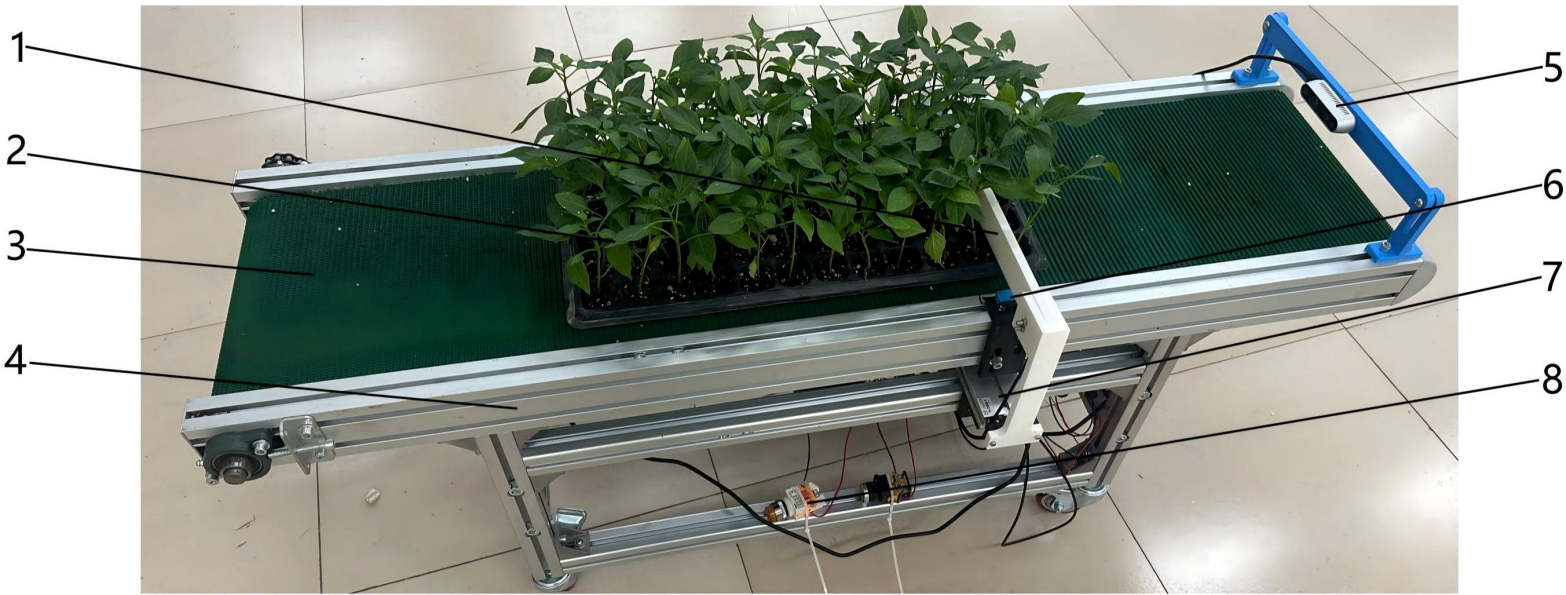
Test bench for precise positioning of pepper seedling clamping points. 1.Shielding-supporting mechanism; 2. Plug pepper seedlings; 3. Conveyor belt; 4. Test stand frame; 5. Depth camera; 6. Diffuse reflection photoelectric switch sensor; 7 Dual-axis cylinder; 8. Electrical control system.

During the visual identification stage, the rear-row seedlings often interfere with the front-row seedlings, adversely affecting the accuracy of main stem recognition in pepper seedlings. To address this issue, a shielding-supporting mechanism was designed based on the morphological characteristics of pepper seedlings. Subsequently, a clamping-point spatial positioning test bench was constructed based on this design, as shown in **Figure 9**. When the conveyor belt delivers the plug pepper seedlings to the designated workstation, a diffuse photoelectric switch sensor detects the signal and transmits it to the PLC. The PLC then controls a dual-axis cylinder to drive the shielding–supporting mechanism to insert between the front-row and rear-row seedlings. During positioning, this mechanism simultaneously performs two functions: supporting the front-row seedlings to prevent excessive tilting and shielding the rear-row seedlings to reduce interference with visual identification.

## 4 Experimental results and analysis

### 4.1 Experimental environment and parameter configuration

The hardware configuration for this experiment consisted of an Intel Xeon Gold 622R CPU and an NVIDIA RTX A4000 GPU. The software environment was based on the Windows 10 operating system, with PyCharm Community Edition 2023 as the development tool. The experiment relied on the PyTorch deep learning framework and was accelerated using CUDA 11.8 and cuDNN 8.0. The programming language used was Python 3.8. The main training parameters are listed in **Table 2**.

**Table 2:** Parameter settings.

Parameter	Value
Input image size	640×640
Cache	True
Batch	32
Epoch	300
Lr0	0.01
Weight decay	0.0005

For system deployment and inference, the trained SRW-YOLOv8n model was executed on a laptop serving as the upper control unit of the clamping-point spatial positioning system. The laptop is equipped with an Intel i7 CPU and 16 GB RAM. The inference and coordinate transformation processes were implemented using the same software framework described above. The model performs real-time main-stem detection, and the detected pixel coordinates are subsequently transformed into spatial coordinates through the calibrated mapping model.

### 4.2 Model performance evaluation metrics

When evaluating the performance of object detection algorithms, different metrics are usually selected according to specific application requirements. To comprehensively assess the detection performance of the constructed SRW-YOLOv8n model in the main-stem detection task of plug pepper seedlings, this study adopts commonly used object detection metrics, including Precision (P), Recall (R), F_1_, and mAP 0.5(Mean Average Precision@0.5), as the primary evaluation indicators.

Among them, P is calculated using Equation 16. It represents the proportion of true positive samples among all samples predicted as positive and reflects the accuracy of the model’s predictions. R is calculated using Equation 17. It represents the proportion of true positive samples that are correctly detected by the model and reflects the model’s detection capability. The F_1_ is calculated using Equation 18. It combines Precision and Recall and is used to evaluate the overall performance of the model with respect to these two metrics.

(16) $$R=\frac{TP}{TP+FN}$$

(17) $$P=\frac{TP}{TP+FP}$$

(18) $$F_{1}=2\frac{PR}{P+R}=\frac{2TP}{2TP+FP+FN}$$

Where, *TP* represents true positive, indicating a correctly detected instance where both the ground truth and the prediction are positive. *FP* represents false positive, indicating a false detection where the ground truth is negative but the prediction is positive. *FN* represents false negative, indicating a missed detection where the ground truth is positive but the prediction is negative.

The mAP represents the average of the Average Precision (AP) values over all classes in an object detection task and is calculated using Equation 19. The mAP@0.5 denotes the mean Average Precision when the Intersection over Union (IoU) threshold is set to 0.5, reflecting the model’s basic object detection capability under relatively lenient conditions. Common evaluation metrics also include mAP@0.5:0.95; however, this metric was not included in this study, as the main-stem detection stage focuses on stem detection rather than clamping-point localization. At an IoU threshold of 0.5, the model is sufficient to satisfy the requirements for accurate main-stem detection of plug pepper seedlings.

(19) $$\text{m}AP=\frac{1}{N}\sum_{i=1}^{N}AP_{i}=\frac{\int_{0}^{1}P(R)\text{d}(R)}{N}$$

Where, *N* represents the number of target classes. In this experiment, there is only one target class; therefore, N equals 1. *AP_i_* represents the Average Precision of the *i*-th target class.

### 4.3 Model training results and analysis

#### 4.3.1 Comparative experiments on dataset partition ratios

**Figure 10:**
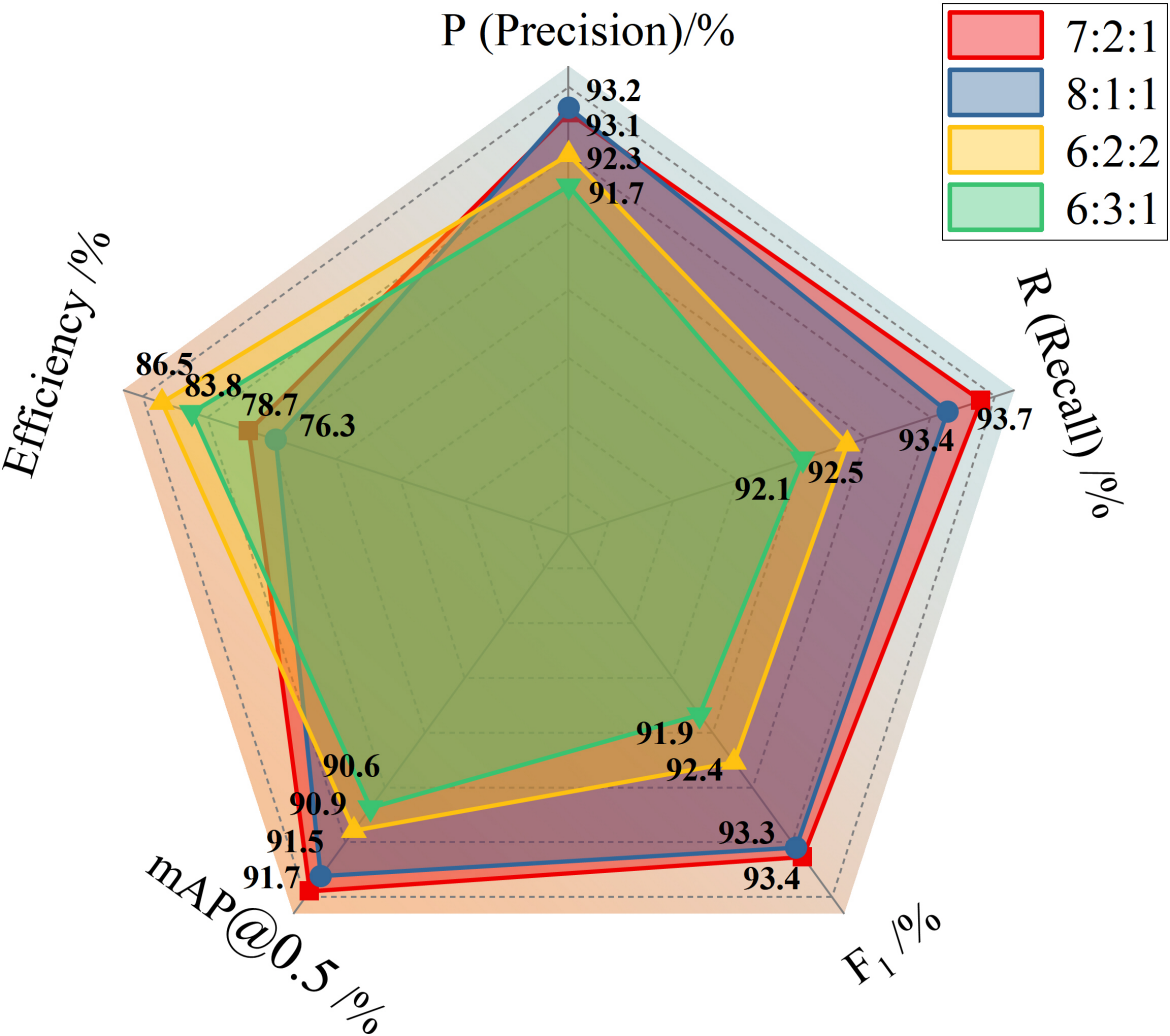
Model training results of four partition ratios.

To explore the optimal partitioning scheme for the self-built plug pepper seedling dataset, this study conducted a comparative experiment on different dataset split ratios. The original YOLOv8n model was used to train and analyze datasets with four different partition ratios. The training results are shown in **Figure 10**.

As shown in **Figure 10**, P, R, F_1_, and mAP@0.5 generally improve as the proportion of the training set increases, reflecting better feature learning with more training data. However, when the training set proportion exceeds 70%, model performance declines. This is likely due to overfitting, where the model memorizes the training samples rather than generalizing to unseen data, and the smaller validation set reduces the reliability of validation metrics for model tuning. Based on this analysis, the 7:2:1 split provides a balance between sufficient training data and reliable validation, ensuring stable model performance. Therefore, this partitioning scheme was adopted in subsequent loss function comparison experiments, ablation studies, and model comparison experiments.

#### 4.3.2 Comparative experiments on loss functions

To verify the effectiveness of the introduced WIoU loss function compared with other commonly used bounding box regression loss functions in the main-stem detection task of plug pepper seedlings, this study conducted a loss function comparison experiment while keeping all other experimental conditions unchanged. The experiment used the CIoU loss function from the original YOLOv8n model as the baseline, replacing only the bounding box regression loss. The experimental results are shown in **Table 3**.

**Table 3:** Comparison experiment results of loss functions.

Loss function	P/%	R/%	F_1_/%	mAP@0.5/%
CIoU	93.1	93.7	93.4	91.7
DIoU	92.8	92.9	92.8	91.2
GIoU	93.5	93.0	93.2	91.6
EIoU	93.3	93.5	93.4	92.6
SIoU	93.6	93.7	93.6	92.3
WIoU	94.3	93.9	94.1	93.8

As shown in **Table 3**, compared with using the original CIoU loss function, the model’s four evaluation metrics all decreased when the DIoU loss function was applied. When the GIoU loss function was used, only P improved by 0.4%, while the other metrics declined. When the EIoU loss function was adopted, P and mAP@0.5 increased by 0.2% and 0.9%, respectively, R decreased by 0.2%, and F_1_ remained unchanged. When the SIoU loss function was employed, P, F_1_, and mAP@0.5 improved by 0.5%, 0.2%, and 0.6%, respectively, while R remained unchanged. Notably, improvements across all four metrics were observed only when the WIoU loss function was applied, with P, R, F1, and mAP@0.5 increasing by 1.3%, 0.2%, 0.7%, and 2.1%, respectively. This improvement is mainly due to the fact that the annotated bounding boxes of plug pepper seedling stems often have similar aspect ratios. In such cases, the aspect-ratio penalty in CIoU becomes ineffective, limiting its ability to optimize bounding box regression. WIoU overcomes this limitation by introducing an explicit size penalty and a dynamic focusing mechanism, allowing the model to better learn subtle differences in stem bounding boxes and achieve higher detection accuracy. In summary, the YOLOv8n model achieved the best performance when using the WIoU loss function on the plug pepper seedling dataset; therefore, this study replaced the original YOLOv8 loss function with WIoU.

#### 4.3.3 Ablation experiment

To systematically evaluate the detection improvements of the proposed optimization strategies compared with the original model, this study designed and conducted a multi-factor ablation experiment, and the results are shown in **Table 4**. With the dataset composition and training hyperparameters kept consistent, the optimization strategies were gradually introduced using a controlled variable approach. Specifically, YOLOv8n was used as the baseline, and the three optimization strategies were sequentially added. By analyzing the model’s performance in terms of P, R, F_1_, mAP@0.5, and parameter count after each introduction, the individual and combined effects of the optimization strategies were assessed.

**Table 4:** Ablation experiment results.

Model number	SimAM	RFAConv	WIoU	P/%	R/%	F_1_/%	mAP@0.5/%	Parameter count
1	×	×	×	93.1	93.7	93.4	91.7	3011043
2	✓	×	×	93.8	94.1	93.9	93.5	3011043
3	×	✓	×	94.2	95.1	94.6	94.3	3042435
4	×	×	✓	94.3	93.9	94.1	93.8	3011043
5	✓	✓	×	95.2	96.3	95.7	95.4	3042435
6	×	✓	✓	94.9	96.2	95.5	95.7	3042435
7	✓	✓	✓	96.6	98.4	97.5	97.4	3042435

As shown in **Table 4**, Models 2, 3, and 4 individually introduced the SimAM, RFAConv, and WIoU loss function. Taking Model 1 (YOLOv8n) as the baseline, Model 2 achieved improvements of 0.7%, 0.4%, 0.5%, and 1.8% in P, R, F1, and mAP@0.5, respectively, without increasing the number of parameters. These findings suggested that introducing the SimAM attention module enhances model performance at no additional computational cost. Model 3 achieved improvements of 1.1%, 1.4%, 1.2%, and 2.6% in P, R, F1, and mAP@0.5, respectively, with only a 1% increase in parameters. This demonstrated that the RFAConv module, through receptive-field-level adaptive weighting, effectively improves detection performance with a minimal increase in model size. Model 4 achieved improvements of 1.2%, 1.2%, 0.7%, and 2.1% in P, R, F1, and mAP@0.5, respectively. This confirmed that the WIoU loss effectively enhances detection accuracy. When both the SimAM and RFAConv modules were simultaneously incorporated in Model 5, the improvements in P, R, F1, and mAP@0.5 reached 2.1%, 2.6%, 2.3%, and 3.7%, respectively. Compared with Model 2 and Model 3, the performance gains were higher than the sum of individual contributions, indicating a positive synergistic effect. Specifically, SimAM first enhances discriminative neuron-level stem features, and RFAConv then adaptively extracts these enhanced features through receptive-field-level weighting. By combining feature enhancement with spatially robust extraction, the two modules complement each other, leading to a synergistic improvement in detection accuracy beyond the sum of their individual contributions. Model 6, which combines the RFAConv module and the WIoU loss function, achieved improvements of 1.8%, 2.5%, 2.3%, and 4.0% in P, R, F1, and mAP@0.5, respectively. These results showed that combining feature-level enhancement (RFAConv) with the more sensitive bounding box regression (WIoU) produces a complementary effect. WIoU effectively refines the localization of features strengthened by RFAConv, leading to more precise detection. Model 7 represents the SRW-YOLOv8n model constructed in this study, which integrates all three improvements. It achieved increases of 3.2%, 4.7%, 4.1%, and 5.7% in P, R, F1, and mAP@0.5, respectively. The performance boost is higher than any partial combination, fully demonstrating the synergistic effect among SimAM, RFAConv, and WIoU. SimAM ensures discriminative neuron-level features, RFAConv robustly extracts these features under occlusion and dense arrangements, and WIoU precisely optimizes bounding box regression. Together, they provide a complementary pipeline that maximizes detection accuracy in challenging scenarios. These findings confirmed that the improvements are not simply additive but interact positively to enhance model performance, fully demonstrating that the SRW-YOLOv8n model outperforms the original YOLOv8 in the main-stem detection task of plug pepper seedlings.

#### 4.3.4 Comparative experiments of different models

To further validate the performance advantages of the constructed SRW-YOLOv8n model proposed in the main-stem detection task of plug pepper seedlings, this study conducted comparison experiments with several mainstream YOLO-series object detection models, including lightweight models such as YOLOv5n, YOLOv7-tiny, and YOLOv8n. The comparative analysis considered both model training performance and actual detection results to comprehensively evaluate the overall performance advantages of the proposed model in this detection task.

In terms of model training performance, under consistent experimental conditions, the algorithm environments for the different models were established, and all models were trained and analyzed using the same dataset. The experimental results are presented in **Table 5**.

**Table 5:** Comparison experiment results of different models.

Model	P/%	R/%	F_1_/%	mAP@0.5/%
YOLOv3-tiny	91.9	92.0	91.9	91.3
YOLOv5n	91.8	92.9	92.3	91.3
YOLOv7-tiny	92.1	92.5	92.3	91.5
YOLOv8n	93.1	93.7	93.4	91.7
YOLOv10n	94.5	94.9	94.7	93.0
SRW-YOLOv8n	96.6	98.4	97.5	97.4

As shown in **Table 5**, in the main-stem detection task of plug pepper seedlings, the SRW-YOLOv8n model demonstrates clear advantages over other lightweight YOLO series comparison models across all performance metrics. Specifically, the F_1_ increased by 5.5%, 5.1%, 5.2%, 4.1%, and 2.8%, respectively, while mAP@0.5 improved by 6.1%, 6.1%, 5.9%, 5.7%, and 5.4%. These results indicated that the proposed detection model has stronger adaptability for detecting seedling main stems in plug tray environments, fully reflecting its performance advantages.

**Figure 11:**
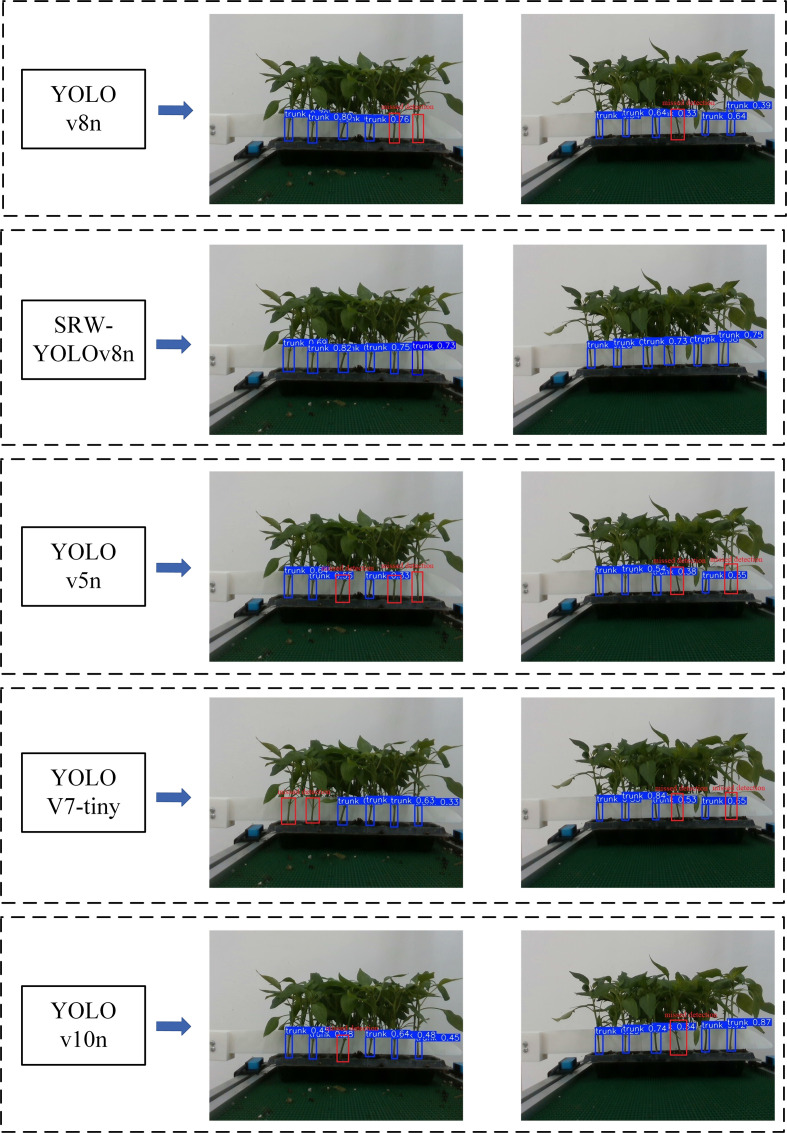
Actual detection results of different models.

In terms of actual detection results, the best training weights (best.pt) obtained from each model were loaded into the detection models under the same experimental conditions. Detection experiments were then conducted on the test set samples. By comparing the missed detections of different models in main-stem detection, the practical detection performance of the models was analyzed. Some of the detection results are shown in **Figure 11**.

As shown in **Figure 11**, the constructed SRW-YOLOv8n model demonstrates superior detection performance for the main stems in the test samples of plug pepper seedlings. In comparison, the other models showed varying degrees of missed detection: YOLOv5n and YOLOv7-tiny each missed 2–3 main stems, while YOLOv8n and YOLOv10n each missed 1–2 main stems. These models are unable to fully adapt to the characteristics of plug pepper seedlings, resulting in lower detection accuracy for small main stems.

Considering both the model training results and the practical detection performance, it is evident that although the detection performance of YOLO series models has steadily improved through successive iterations, simply relying on model version upgrades is insufficient to fully meet the detection requirements for plug pepper seedlings. The constructed SRW-YOLOv8n model, with task-specific structural improvements and optimizations, outperformed existing lightweight YOLO models in both detection accuracy and practical application performance, further demonstrated its effectiveness and advancement in the main-stem detection task of plug pepper seedlings.

### 4.4 Training results and analysis of the SRW-YOLOv8n model

**Figure 12:**
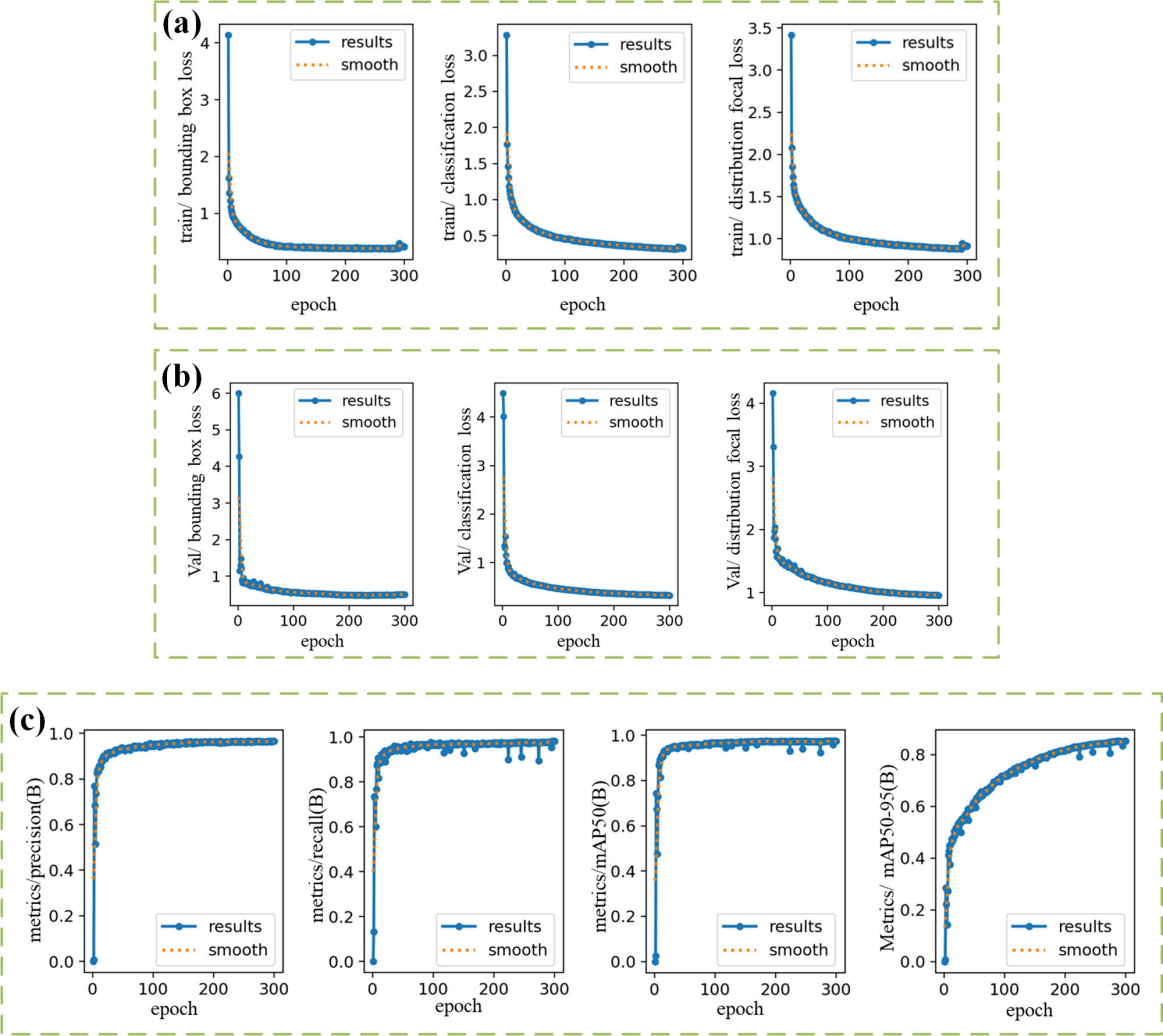
Training curve of SRW-YOLOv8n model. **(a)** Training loss curves; **(b)** Validation loss curves; **(c)** Precision, Recall, and mAP curves.

To further analyze the training characteristics and performance of the SRW-YOLOv8n model in the main-stem detection task of plug pepper seedlings, this study conducted a systematic analysis based on the training curves shown in **Figure 12**. The analysis focuses on the convergence behavior during training and the evolution of detection performance with increasing training epochs. This is done to verify the stability and reliability of the model.

As shown in **Figure 12a** and **Figure 12b**, the loss functions of the SRW-YOLOv8n model on both the training set and the validation set exhibit good convergence as the number of epochs increases. In the early stage of training, all loss values decrease rapidly within the first 100 epochs and reach an initial convergence. Around 200 epochs, the loss curves become stable, with no obvious oscillation or divergence. As shown in **Figure 12c**, the curves of P, R, and mAP@0.5 gradually stabilize after approximately 100 epochs, while the mAP@0.5:0.95 metric continues to increase. This indicates that the localization accuracy of the model under higher IoU thresholds is still improving.

Considering both the convergence characteristics and the variation trends of detection performance, the optimal weight model obtained after 300 training epochs (best.pt) was selected for subsequent main-stem detection experiments of plug pepper seedlings.

### 4.5 Camera calibration

**Figure 13:**
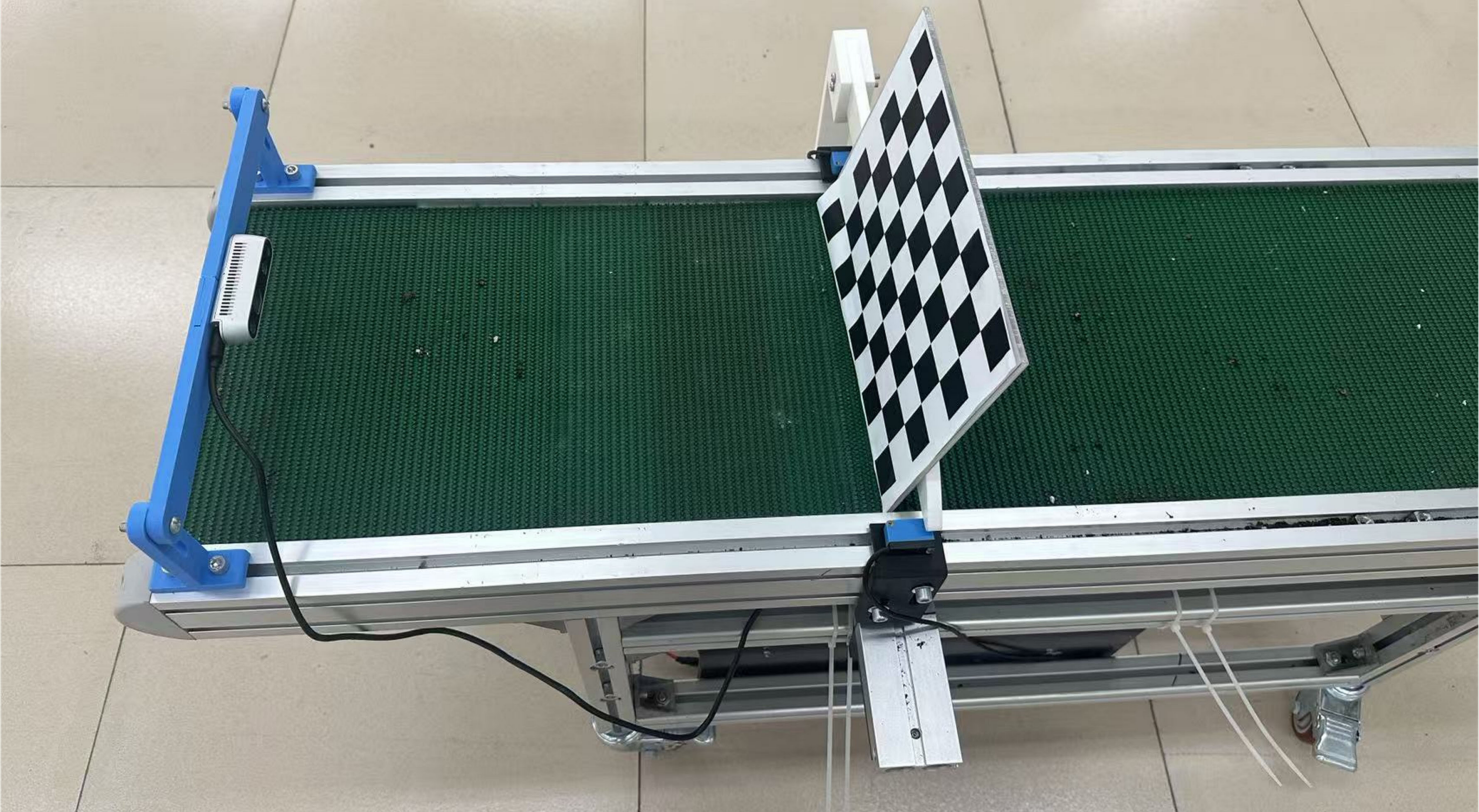
Chessboard-based camera calibration.


Camera calibration is a prerequisite for the precise spatial positioning of clamping-points in plug pepper seedlings. The objective of camera calibration is to obtain the camera’s intrinsic parameters and distortion coefficients. The intrinsic parameters describe the imaging characteristics of the camera, including the focal length and the principal point, while the distortion coefficients are used to correct radial and tangential lens distortions, thereby improving imaging accuracy. In this study, the Zhang’s calibration method was employed to calibrate the RealSense D435i depth camera (as shown in **Figure 13**). By capturing multiple images of a chessboard calibration pattern, the camera’s intrinsic matrix K and distortion coefficients M were obtained.

$$\begin{array}{l} \text{K}=\left[\begin{array}{ccc} f_{x} & 0 & c_{x}\\ 0 & f_{y} & c_{y}\\ 0 & 0 & 1 \end{array}\right]=\left[\begin{array}{ccc} 582.09 & 0 & 310.66\\ 0 & 583.48 & 212.01\\ 0 & 0 & 1 \end{array}\right]\\ \text{M}=\left[\begin{array}{cccc} -0.04 & 1.22 & -0.17 & \begin{array}{cc} 0.01 & -3.61 \end{array} \end{array}\right] \end{array}$$

### 4.6 Clamping-point positioning results and analysis

#### 4.6.1 Clamping-point image localization

**Figure 14:**
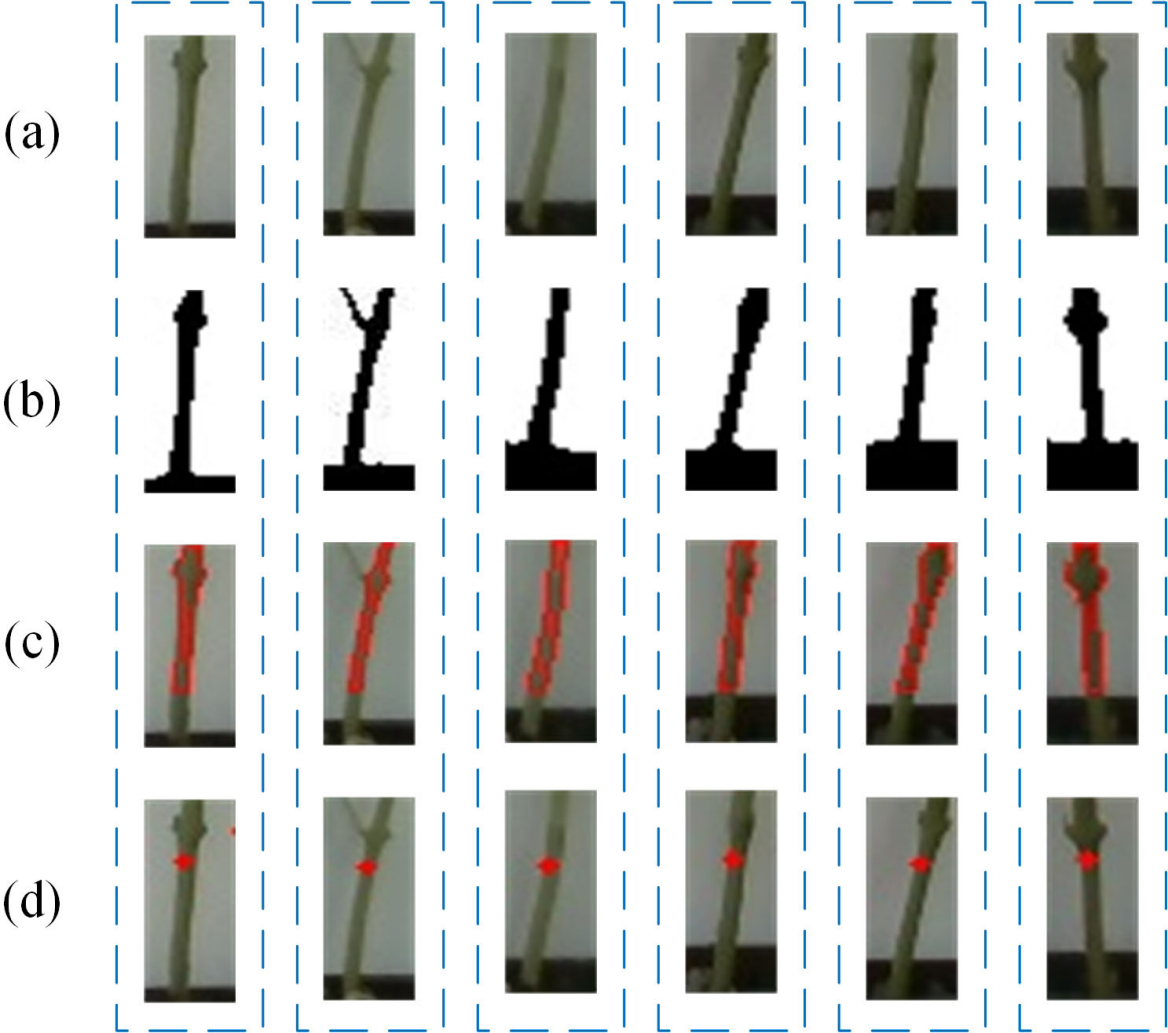
Structured image processing results of detection bounding boxes for a row of pepper seedlings. **(a)** Detection bounding box image; **(b)** Binary image; **(c)** Contour extraction image; **(d)** Clamping-point image.

The purpose of clamping-point image localization is to obtain the pixel coordinates of the clamping-point, providing pixel-level information for subsequent coordinate transformation. After extracting the detection bounding boxes of the main stems of an entire row of plug pepper seedlings, the structured image processing pipeline proposed in this study is applied to the images within the bounding boxes to obtain the image location of the clamping-point. The results are shown in **Figure 14**.

As illustrated in **Figure 14**, the proposed structured image processing pipeline successfully localizes the image positions of clamping-points for an entire row of plug pepper seedlings, demonstrating strong robustness and adaptability to seedlings with varying morphological characteristics. When applied to pepper seedlings with relatively thin main stems, the traditional YOLOv8 keypoint detection method often located target points outside the main stem, resulting in large detection errors and reduced spatial positioning accuracy. In contrast, the proposed method effectively avoids this issue, significantly improving clamping-point localization accuracy and ensuring the reliability of subsequent spatial positioning.

#### 4.6.2 Clamping-point spatial positioning

After obtaining the image positions of the clamping-points of the pepper seedlings, a coordinate transformation method is used to map the pixel coordinates to three-dimensional spatial coordinates in the camera coordinate system, which are then compared with the actual positions. The results are shown in **Table 6**.

**Table 6:** Spatial positioning results and errors of clamping points for pepper seedlings.

Seedling serial number	Machine vision positioning coordinates			Actual position coordinates			Absolute error		
	x/mm	y/mm	z/mm	x′/mm	y′/mm	z′/mm	x/mm	y/mm	z/mm
1	-93.91	37.01	400.00	-91.50	34.50	397.50	2.41	2.51	2.50
2	-46.15	45.58	403.00	-44.00	48.00	401.50	2.15	2.42	1.50
3	1.60	38.97	399.00	-1.00	37.00	402.00	2.60	1.97	3.00
4	37.02	42.92	404.00	39.50	40.50	405.00	2.48	2.42	1.00
5	95.80	44.15	409.00	93.00	47.00	407.50	2.80	2.85	1.50
6	132.39	37.66	407.00	130.00	35.50	408.50	2.39	2.16	1.50
Average							2.47	2.39	1.83

As shown in **Table 6**, the proposed spatial positioning method for clamping-points of plug pepper seedlings achieves average absolute positioning errors of 2.49 mm, 2.39 mm, and 1.83 mm along the x, y, and z axes, respectively. The relatively smaller error along the z-axis indicated that the depth-based spatial mapping is highly stable, which can be attributed to the direct measurement capability of the depth camera along the optical axis. In contrast, the errors along the x and y axes are relatively larger. Analysis showed that the limitation of image resolution on pixel coordinate precision is the dominant source of error, followed by cumulative effects from camera calibration, while contour extraction errors generally contribute less. Overall, the proposed method maintains good stability while ensuring high positioning accuracy, meeting the precision requirements for clamping operations of plug pepper seedlings.

### 4.7 Clamping-point spatial positioning system

**Figure 15:**
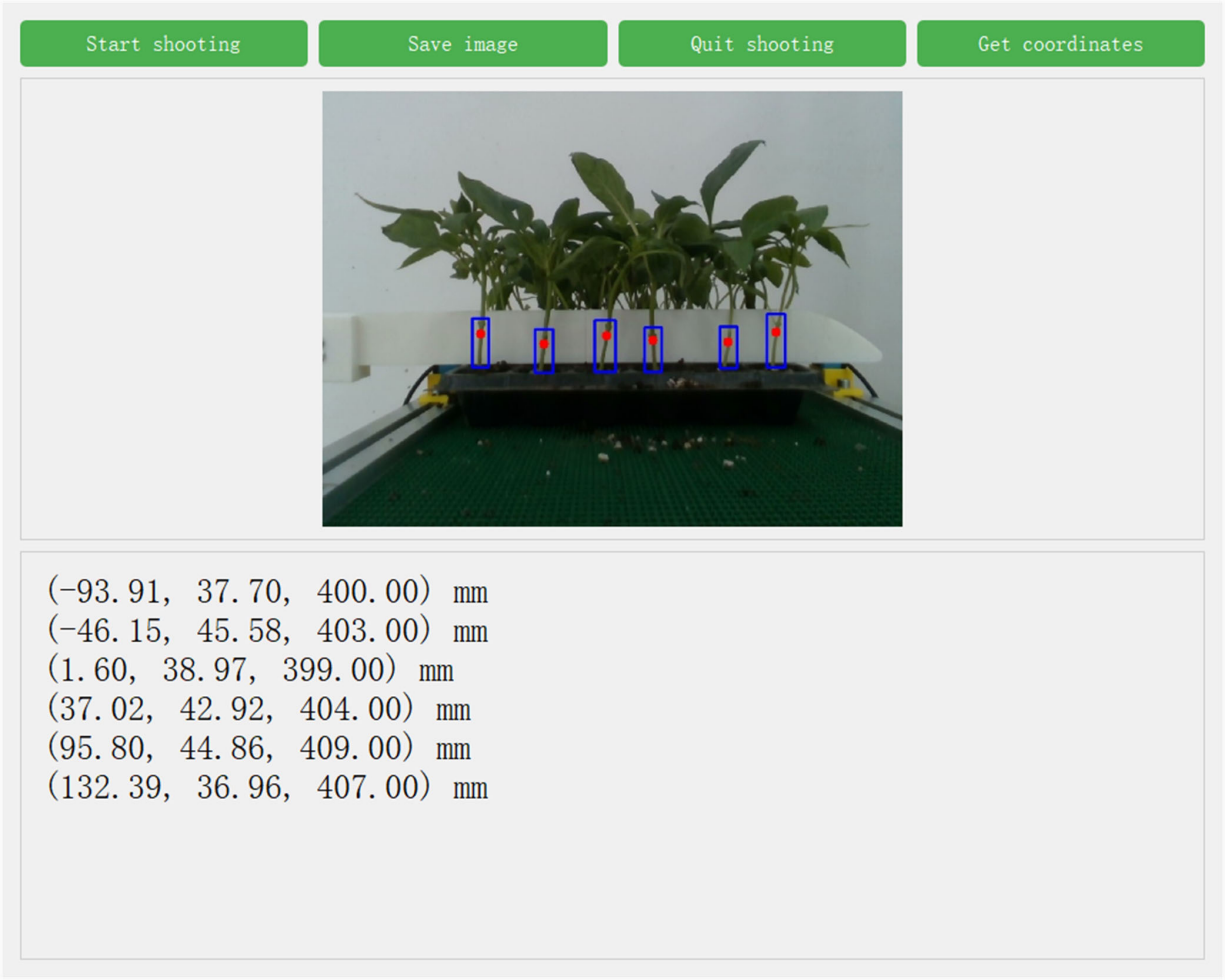
Clamping point spatial positioning system interface.

To support practical engineering applications, a visual localization system integrating main-stem detection and clamping-point spatial positioning was developed for plug pepper seedlings. The graphical user interface (GUI) of the system was developed using the Qt framework, and the system was deployed on the experimental platform described in Section 4.1. The overall interface, functional architecture, and example operational results of the system are illustrated in **Figure 15**.

During system operation, color and depth images of plug pepper seedlings are synchronously acquired using the Intel RealSense D435i depth camera, and the captured data are transmitted to the host computer for subsequent processing. On the upper computer, the trained SRW-YOLOv8n recognition model is employed to detect the main-stem region of each seedling in an entire row of the plug tray. Based on the detected bounding boxes, the proposed structured image processing pipeline is further applied to accurately determine the pixel coordinates of the clamping points. After obtaining the pixel-level localization results, camera calibration parameters and the established coordinate transformation model are used to convert the pixel coordinates into 3D spatial coordinates in the camera coordinate system.

## 5 Conclusion

This study presents a spatial positioning method for clamping-points of plug pepper seedlings based on the SRW-YOLOv8n object detection model. First, the SRW-YOLOv8n model was constructed by improving the YOLOv8 model. Experimental results demonstrated that the constructed model achieved improvements of 3.2%, 4.7%, 4.1%, and 5.7% in P, R, F_1_ and mAP@0.5, respectively, enabling more reliable main-stem detection. Based on main-stem detection, the proposed method combines a structured image processing pipeline to accurately extract the pixel coordinates of clamping-points, and obtains their three-dimensional spatial coordinates through a depth camera and coordinate mapping. In addition, a shielding–supporting mechanism was designed, and a spatial localization test platform for plug pepper seedlings was established to verify the accuracy and stability of the proposed method. Spatial positioning experiments showed that the average absolute positioning errors in the x, y, and z directions were 2.49 mm, 2.39 mm, and 1.83 mm, respectively, fully meeting the accuracy requirements for grafted seedling collection. In summary, the proposed method significantly improves the accuracy of main-stem detection and clamping-point positioning, providing reliable technical support for key operations in pepper seedling grafting.

## 6 Limitations and future work

The current study focuses solely on the visual detection and spatial positioning of clamping points for plug pepper seedlings. The physical clamping mechanism and closed-loop robotic execution have not yet been integrated, which limits the system to coordinate generation rather than complete automated grafting or picking operations.

Future work will focus on integrating the visual localization system with the physical clamping mechanism to achieve fully automated clamping and grafting operations. Further improvements will include optimizing the algorithm to enhance robustness under diverse environmental conditions, extending the system to accommodate a wider range of pepper varieties and seedling growth stages, and exploring multi-camera setups or mobile platforms to increase operational flexibility and scalability for practical production environments.

## Data Availability

The original contributions presented in the study are included in the article/supplementary material. Further inquiries can be directed to the corresponding author.
